# Fauna and conservation in the context of formal education: a study of urban and rural students in the semi-arid region of Brazil

**DOI:** 10.1186/s13002-020-00374-4

**Published:** 2020-04-25

**Authors:** José Valberto de Oliveira, Moacyr Xavier Gomes da Silva, Anna Karolina Martins Borges, Wedson Medeiros Silva Souto, Sérgio de Faria Lopes, Dilma Maria de Brito Melo Trovão, Raynner Rilke Duarte Barboza, Rômulo Romeu Nóbrega Alves

**Affiliations:** 1 Departamento de Biologia e Programa de Pós-Graduação em Etnobiologia e Conservação da Natureza, Universidade Estadual da Paraíba, Av. das Baraúnas, 351/Campus Universitário, Bodocongó, Campina Grande, PB 58109-753 Brazil; 2 Programa de Pos-Graduação em Ciências Biológicas (Zoologia), Departamento de Sistemática e Ecologia da Universidade Federal da Paraíba, João Pessoa, PB 58051-900 Brazil; 3 Department of Biology, Laboratory of Zoology, Wildlife Use and Conservation (ZUCON), Federal University of Piaui (UFPI), Campus Ministro Petrônio Portella, Teresina, PI 64049-550 Brazil; 4 Departamento de Biologia, Universidade Estadual da Paraíba, Av. das Baraúnas, 351/Campus, Universitário, Bodocongó, Campina Grande, PB 58109-753 Brazil; 5 Colégio Aplicação - CAP, Universidade Federal de Roraima, Avenida Capitão Ene Garcês, 2413 - Aeroporto, Campus do Paricarana, Boa Vista, 69310-000 Brazil

**Keywords:** Wild fauna, Biological education, Ethnozoology, Conservation of nature

## Abstract

**Background:**

In addition to playing a key role in the dynamics of ecosystems, animal diversity, especially that of wild vertebrates, is intimately linked with human evolutionary history, which has resulted in diverse interactions that must be emphasized in formal education processes. We analyzed several methods of approaches used for biological education in order to teach about wild vertebrates and their conservation in urban and rural schools in the semi-arid region of Brazil.

**Methods:**

Data were obtained via questionnaires applied to 990 students, of which 528 were urban and 462 rural, distributed among the seven grades/years that comprise the last two cycles of basic education in Brazil. The richness and diversity of the animals cited by the students were calculated, being the diversity using an adaptation of the equation for the Shannon-Weaver Index (*H*′). Data were analyzed using non-parametric descriptive statistics.

**Results:**

Mammals and birds had the greatest richness and diversity of animals cited as most-studied in science/biology classes, and also the most indicated as occurring in the studied region. Among mammals, large carnivores with a showy appearance and utilitarian value had the highest citation frequencies, while there was a tendency for limited recognition of faunistic diversity in the other groups mentioned. Almost 70% of the students stated that their schooling processes dealt with the conservation of wild animals; however, about 50% of the students in both urban and rural contexts did not express conceptual understanding about the conservation of nature.

**Conclusions:**

The recognition of animal diversity, especially vertebrates, beyond just mammals and birds, as well as conceptual clarity about the conservation of nature, are fundamental factors for the development of critical awareness of fauna and its conservation, and where the processes of schooling have a preponderant role. Finally, the study contributes to the legitimization of Ethnobiology as an interdisciplinary field of knowledge, especially in its interface with education, in addition to pointing out the importance of optimizing efforts in approaches to biodiversity conservation in formal educational processes.

## Background

Knowledge of biological diversity and its conservation must be fundamental requirements of public education, with the aim of developing critical awareness regarding the relationships/interactions between humans and other living beings [1–5]. Among the array of biological diversity, wild vertebrates are perhaps the group that has been most involved with human evolutionary history, being protagonists of important activities, such as hunting and domestication [6]. In addition, vertebrates play important roles in relationships/interactions within ecosystems, which reinforce the relevance of addressing them from the point of view of animal conservation in formal education processes [1–5].

According to Brazilian curricular guidelines for basic education, the study of animals should be undertaken in the context of “thematic blocks” (e.g., “Environment” and “Life and Environment”) at the Ensino Fundamental level/Elementary School (grades 1st–9th), and “structuring themes” (e.g., “interactions between living beings” and “diversity of life”) at the Ensino Médio level/High school (grades 1st–3rd), from an ecological and evolutionary perspective. Moreover, it should facilitate internal approaches to curricular cycles, with emphasis on contextualizations, connections between thematic blocks, connections between structuring themes, and connections with other areas and “transversal themes.” The guidelines thus suggest an interdisciplinary approximation of approaches with the aim of developing a critical conscience that guides human postures and values in relationships with nature [1–5].

In the process of curricular evolution, the expanded diversification of studied living organisms aims, mainly, to develop valorization of biodiversity and the preservation of environments [1, 2]. This perspective converges with the educational objectives of biodiversity education, by emphasizing the importance of diversifying known organisms, with schooling having a fundamental role [7]. Educational approaches need to go beyond the curricular tradition of schemes and classifications based on systematics, watertight descriptions and decontextualized, and move toward more phylogenetic, ecological, and, above all, contextual bases observable directly or indirectly in real environments. Furthermore, animals need to be considered as to which are native or introduced to regions, which are economically significant and why, and which are threatened with extinction and why [2].

Previous studies involving relationships between students and animals highlight some issues related to the aforementioned perspectives, including school learning about fauna that tends to be dissociated from real situations, contributing to conceptual misunderstanding and limited knowledge [6]; science and biology curricula should include some content related to local biodiversity, with more emphasis on endangered species, and not be limited to exotic animals [8]; and formal education, in general, devotes little time to the study of biodiversity, which occurs almost exclusively by means of information through decontextualized schemes and images [7]. Therefore, the search for a conceptual understanding of biodiversity and its conservation needs a curriculum that is based on a more contextual, systematic, and interdisciplinary perspective [9].

Brazilian curricular guidelines also highlight an emphasis on interactions and correlations between thematic axes, as well as transversal themes such as “Environment” and its contents. They suggest that “Environment” permeate all education practice by way of “Environmental Education” (EA), with the aim of developing critical awareness and a sense of individual and collective responsibility at local, national, and planetary levels, prioritizing a didactic movement from local to global and vice versa [10, 11].

Among the conceptual notions foreseen in the transversal theme “Environment,” we highlight “conservation,” “sustainability,” and “biodiversity.” “Conservation” expresses the use of resources of nature in a “rational” way with the aim of good yield without exhausting capacity for “renewal” or “self-sustenance”; from the point of view of Brazilian legislation, this “involves managing, using with care, and maintaining” [10, 11]. The notion of “sustainability” presupposes the satisfaction of present needs, without compromising the needs of future generations; that is, “to improve the quality of human life within the limits of the support capacity of ecosystems.” The first two conceptual notions necessarily converge to a third—biodiversity; that is, the sustainability of life in all its forms, including human life, fundamentally presupposes the conservation of biodiversity, as understood to be the total set of “genetic availability of different species and varieties, of different ecosystems” [10, 11]. These concepts converge with theoretical orientations of ecology [12–16].

Beyond its recognized importance, the conservation of biodiversity is a matter of ethical principle since all living humans belong to the same unique network of planetary life. It should be noted that, in this context, the application of such an understanding also refers to the notion of cultural diversity since these two dimensions—biological and cultural—are two faces of the same coin in social imagination and in practical life, and interact with, and thus influence, one another, which confers them dynamicity, change, and evolution [10, 11]. From this perspective, the role of contemporary schooling is inserted, for instance, in the convergence of cultural diversity, in order to make possible the reconstruction and/or rearrangement of humans perceptions, conceptions, concepts, and demystifications from the scientific culture experienced in it, as suggested by Alves et al. [17], about ethnobiological approaches in the establishment of relationships between local and scientific knowledge in the school environment, in the face of strategies of the conservation of fauna.

In Ensino Médio level, which configures the three final grades of basic education in Brazil (grades 1st–3rd), the evolutionary-ecological approach is reiterated for the study of biodiversity, including fauna [3]. The objective is to understand nature “as an intricate network of relationships” and a complex dynamic, which includes humankind, as well as the diversity of species, as a consequence of the evolutionary process, considering temporal and spatial dimensions. Among the competencies and abilities to be developed in the context of “representation and communication,” is, among others, the ability to describe the characteristics and processes of observed environments or living beings. In the context of “research and understanding,” the expectation is to classify animals, for example, on the basis of scientific criteria while in the “socio-cultural” context, the expectation is to identify the interference of mystical determinants and cultural common sense in relation to aspects of biology. Several studies have emphasized the implications of bio-ecological knowledge for the demystification and qualification of human interactions with other animals [6, 7, 17, 18].

Among the challenges facing biological education is the importance of situating the learner as a participant in contemporary debates. This involves instilling a consciousness of being a citizen of a country that is home to some of the greatest biodiversity on the planet (Brazil) and understanding it beyond simply the number of species but in all its levels, including ecosystems, populations, species, and genes.

Also, the learner should include on their educational process ecological, taxonomic, and genetic aspects; as well as the uses and products originating from the biodiversity, the so-called “environmental services.” Thus, the student will be able to position himself with ethical and scientific coherence in the defense of the conservation of the ecosystem and the biodiversity where it is inserted [5].

In view of these considerations, the present study aimed to analyze the educational approach used concerning fauna (especially wild vertebrates) and their conservation for students of urban and rural schools in the semi-arid region of Brazil. The study was guided by the following questions: (1) Which animals are the most studied in the educational processes experienced by students? (2) Is there discussion about vertebrates of the region being studied in the educational processes experienced by students? (3) For affirmative answers, which animal groups are most represented in terms of richness and diversity in urban and rural realities? (4) Was the importance of wildlife conservation emphasized in the educational processes experienced? (5) What understanding do students express about “conservation of nature”? (6) Are there differences in this understanding between students in Ensino Fundamental (years 1–9) and those in Ensino Médio (years 10–12)?

## Methods

### Study area

**Fig. 1:**
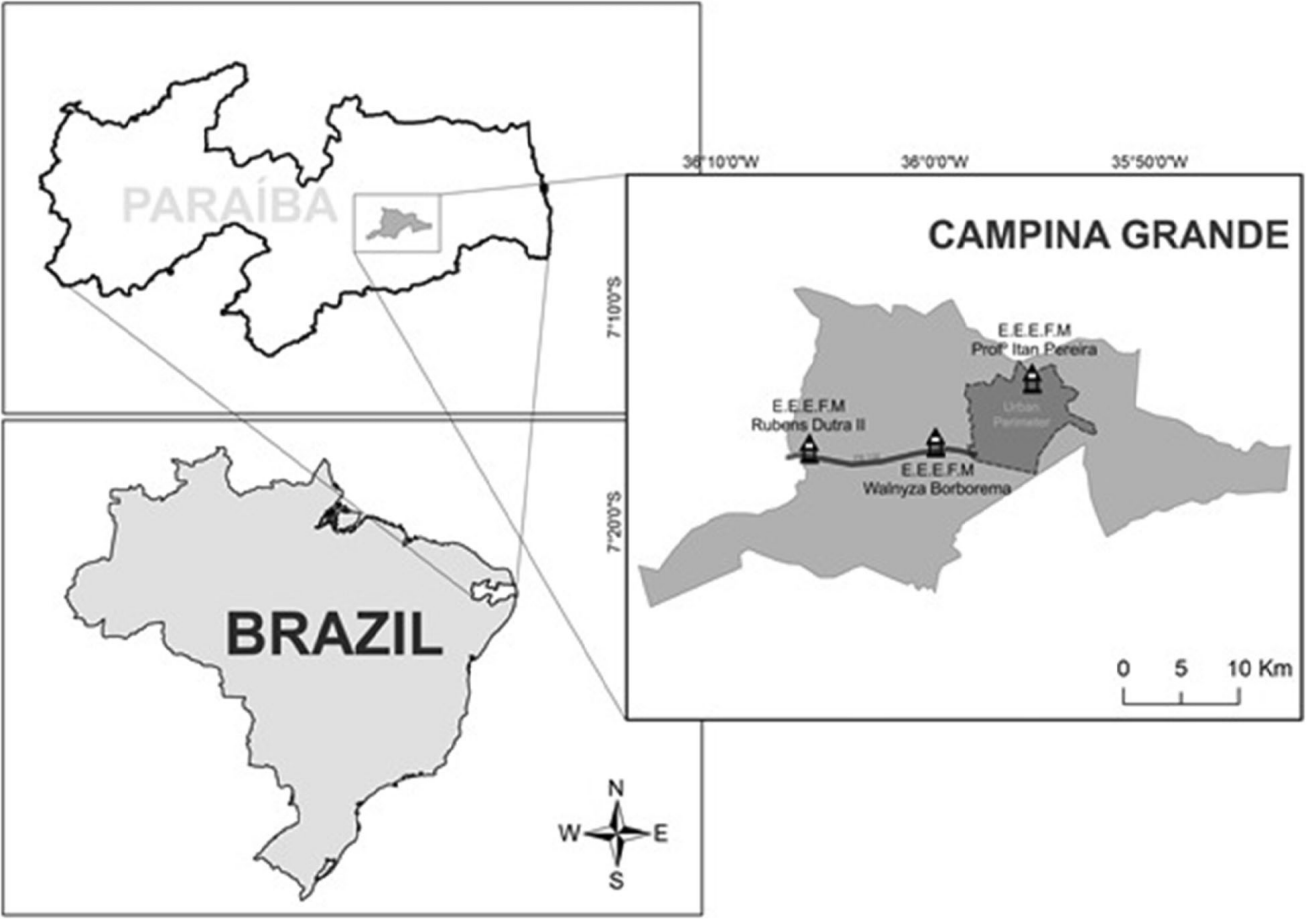
Map showing locations of the studied schools in the municipality of Campina Grande, Paraíba, Brazil

The research was carried out in three schools—one urban and two rural—of the state network in the municipality of Campina Grande (07° 13′ 50′′ S, 35° 52′ 52′′ W), state of Paraíba, Northeast Brazil (Fig. 1). Campina Grande encompasses an area of 593.026 km^2^ with a population of 385,213 inhabitants, 367,209 of which are urban and 18,004 of which are rural, with a density of 648.31 inhabitants/km^2^. The municipality has a Human Development Index (HDI) of 0.720 [19].

School units were selected that had complete Fundamental II and Médio levels (grades 6th–9th and 1st–3rd, respectively) of education. In the studied area, rural education is concentrated in schools located at strategic points (e.g., districts) where students are provided access by public school transportation. Two rural schools were included with the aim of making the sample size (*n*) of urban and rural students more similar.

The following schools were selected according to the criteria described above: (1) Escola Estadual de Ensino Fundamental e Médio Professor Itam Pereira, created by Decree No. 21.039/2000 and located in the urban west area of the municipality; (2) Escola Estadual de Ensino Fundamental e Médio Rubens Dutra Segundo, created by Decree No. 13.151/1989 and located in Distrito de Catolé de Boa Vista, 26 km west of the center of the municipality and accessed by highway BR 230; and (3) Escola Estadual de Ensino Fundamental e Médio Walnyza Borborema Cunha Lima created by Resolution No. 36.730/2006/2016, and located in Sítio Estreito, 12 km west of the center of the municipality and also accessed by BR 230 (Fig. 1). At the time of the study, the schools had 942, 328, and 444 students enrolled, respectively, among the regular and special education programs. Among these students, the study included all those enrolled in the seven grades—6th to 9th of Ensino Fundamental II and grades 1st to 3rd of Ensino Médio—which correspond to the last two regular cycles of basic education in Brazil.

## Methodological procedures

### Data collection

Data were collected through semi-structured questionnaires applied to 990 students: 528 urban and 462 rural, 526 females and 464 males, ages ranging from 9 to 38 years. The questionnaires were given between June and October 2015 to 24 classes of Ensino Fundamental II (grades 6th–9th) and 14 classes of Ensino Médio (grades 1st–3rd), for a total of 38 science/biology classes. The structure of the questions suggested the following actions to the students: express by nomination the animals most studied in science/biology classes; indicate if the classes addressed vertebrates of the region and if so what were they; express whether there was discussion about the conservation of wild animals in science/biology classes; and present your understanding of “Conservation of Nature.”

The research was approved by the Comitê de Ética em Pesquisa (Committee of Ethics in Research) of Universidade Estadual da Paraíba (Protocol CEP-UEPB: 43589815.0.0000.5187). Data collection was proceeded by contacting the administration of the teaching units to acquire authorization for carrying-out the research. After these procedures, and agreement by the respective science/biology faculty, the purposes of the investigation were presented to the students and the “Termos de Consentimento Livre e Esclarecidos–TCLEs” (Terms of Free and Informed Consent), an ethical/legal condition to participate in the research, was forwarded to their parents and/or guardians. Only after the receipt of the properly signed TCELs were questionnaires initiated in the classroom.

### Data analysis

Richness values were calculated for animals cited as the most studied in science/biology classes and as occurring in the region. The diversity 1of the animals cited in both situations was calculated using an adaptation of the equation of the Shannon-Weaver Index (*H*′) [20], in which ni = number of citations for the ith animal; *N* = total number of citations; *S* = total number of animals cited; and ln = Napierian logarithm.

$$ {H}^{\prime }=\frac{\left[N\ln (N)-\sum \limits_{i=1}^S{n}_i\ln \left({n}_i\right)\right]}{N} $$

The data were initially tested for normality using the Shapiro-Wilk test and for homoscedasticity by the Levene test. The obtained data were then analyzed using nonparametric descriptive statistics. The general data were organized in tables as percentages using the program Past 2.17c [21].

## Results

Among the animals indicated by the students as the most studied in science/biology classes, mammals had the highest richness and diversity followed by birds for both the urban (*n* = 34 mammals, *H*′ = 2.737; *n* = 13 birds; *H*′ = 1.750) and rural (*n* = 30 mammals, *H*′ = 2.673; *n* = 14 birds, *H*′ = 1.918) students. High diversity was also observed for invertebrates, despite the low “species” richness cited compared to other animal groups, for both urban (*n* = 12 invertebrates, *H*′ = 1.694) and rural (*n* = 20 invertebrates, *H*′ = 2.400) students (see Table 1). With the exception of fish for rural students, and amphibians for urban students, the average number of citations at the end of Ensino Médio was greater than those at the end of Ensino Fundamental II for all situation analyzed (see Table 2).

**Table 1:** Number of citations of animals in groups, and their respective frequencies, by research participants as the most studied animals in science/biology classes

Group	Taxon	Animal^a^	General	Urban	Rural
Fish	Superclass Pisces	Fish	104 (89.65)	61 (88.40)	43 (91.48)
	Class Selachimorpha	Shark	8 (6.89)	5 (7.24)	3 (6.38)
	Order Gasterosteiformes	Seahorse	3 (2.58)	2 (2.89)	1 (2.12)
	Order Osteoglossiformes	Piranha fish	1 (0.86)	1 (1.44)	0 (0.00)
Total	4	4	116 (99.98)	69 (59.48)	47 (40.51)
Amphibians	Class Amphibia	Amphibians	26 (30.58)	11 (23.91)	15 (38.46)
	Order Anura	Toad	47 (55.29)	25 (54.34)	22 (56.41)
		Frog	5 (5.88)	3 (6.52)	2 (5.12)
		Tree-frog	5 (5.88)	5 (10.86)	0 (0.00)
	Order Caudata	Salamander	2 (2.35)	2 (4.34)	0 (0.00)
Total	3	5	85 (99.98)	46 (54.11)	39 (45.88)
Reptiles	Class Reptilia	Reptiles	39 (21.91)	18 (18.36)	21 (26.25)
	Order Squamata	Snake	106 (59.55)	56 (57.14)	50 (62.50)
		Lizard	7 (3.93)	6 (6.12)	1 (1.25)
		Chameleon	1 (0.56)	1 (1.02)	0 (0.00)
		Tegu lizard	1 (0.56)	0 (0.00)	1 (1.25)
		Gecko	5 (2.80)	3 (3.06)	2 (2.50)
	Order Chelonia	Tortoise	5 (2.80)	3 (3.06)	2 (2.50)
	Order Crocodylia	Alligator	8 (4.49)	7 (7.14)	1 (1.25)
	Order Saurischia	Dinosaur	6 (3.37)	4 (4.08)	2 (2.50)
Total	5	9	178 (99.97)	98 (55.05)	80 (44.94)
Birds	Class Aves	Birds	61 (50.41)	35 (52.23)	26 (48.14)
		Wild birds	9 (7.43)	6 (8.95)	3 (5.55)
	Order Psittaciformes	Blue Macaw	1 (0.82)	0 (0.00)	1 (1.85)
		Parakeet	3 (2.47)	3 (4.47)	0 (0.00)
		Parrot	3 (2.47)	2 (2.98)	1 (1.85)
	Order Accipitriformes	Vulture	4 (3.30)	3 (4.47)	1 (1.85)
	Order Piciformes	Woodpecker	1 (0.82)	1 (1.49)	0 (0.00)
		Toucan	2 (1.65)	1 (1.49)	1 (1.85)
	Order Struthioniformes	Greater rhea	3 (2.47)	2 (2.98)	1 (1.85)
	Order Columbiformes	Ground-dove	3 (2.47)	0 (0.00)	3 (5.55)
		Eared-dove	1 (0.82)	0 (0.00)	1 (1.85)
	Order Apodiformes	Hummingbird	5 (4.13)	0 (0.00)	5 (9.25)
	Order Falconiformes	Hawk	12 (9.91)	8 (11.94)	4 (7.40)
	Order Cuculiformes	Greater Ani	1 (0.82)	1 (1.49)	0 (0.00)
	Order Pelecaniformes	Heron	2 (1.65)	1 (1.49)	1 (1.85)
	Order Strigiformes	Owl	7 (5.78)	3 (4.47)	4 (7.40)
	Order Galliformes	Domestic Hen	3 (2.47)	1 (1.49)	2 (3.70)
Total	12	17	121 (99.89)	67 (55.37)	54 (44.62)
Mammals	Class Mammalia	Mammals	156 (22.94)	79 (20.73)	77 (25.75)
	Order Carnivora	Lion	83 (12.20)	53 (13.91)	30 (10.03)
		Bear	13 (1.91)	11 (2.88)	2 (0.66)
		Jaguar	38 (5.58)	22 (5.77)	16 (5.35)
		Tiger	27 (3.97)	23 (6.03)	4 (1.33)
		Fox	7 (1.02)	4 (1.04)	3 (1.00)
		Wolf	1 (0.14)	1 (0.26)	0 (0.00)
		Maned wolf	2 (0.29)	1 (0.26)	1 (0.33)
		Leopard	1 (0.14)	0 (0.00)	1 (0.33)
		Wild cat	1 (0.14)	0 (0.00)	1 (0.33)
		Dog	65 (9.55)	39 (10.23)	26 (8.69)
		Cat	46 (6.76)	29 (7.61)	17 (5.68)
		Seal	1 (0.14)	1 (0.26)	0 (0.00)
		Otter	1 (0.14)	1 (0.26)	0 (0.00)
		Ruminants	1 (0.14)	1 (0.26)	0 (0.00)
	Family Felidae	Felines	1 (0.14)	1 (0.26)	0 (0.00)
	Order Proboscidea	Elephant	13 (1.91)	8 (2.09)	5 (1.67)
	Order Cetacea	Dolphin	2 (0.29)	2 (0.52)	0 (0.00)
		Whale	35 (5.14)	21 (5.51)	14 (4.68)
	Order Chiroptera	Bat	21 (3.08)	13 (3.41)	8 (2.67)
	Order Pilosa	Anteater	5 (0.73)	2 (0.52)	3 (1.00)
		Sloth	1 (0.14)	0 (0.00)	1 (0.33)
	Order Cingulata	Armadillo	1 (0.14)	0 (0.00)	1 (0.33)
		Brazilian three-banded armadillo	1 (0.14)	1 (0.26)	0 (0.00)
	Order Perissodactyla	Horse	19 (2.79)	7 (1.83)	12 (4.01)
		Zebra	4 (0.58)	1 (0.26)	3 (1.00)
	Order Cetartiodactyla	Deer	7 (1.02)	5 (1.31)	2 (0.66)
	Order Rodentia	Rat	22 (3.23)	15 (3.93)	7 (2.34)
		Guinea pig	2 (0.29)	1 (0.26)	1 (0.33)
		Capybara	5 (0.73)	3 (0.78)	2 (0.66)
		Hamster	1 (0.14)	0 (0.00)	1 (0.33)
	Order Primates	Monkey	27 (3.97)	12 (3.14)	15 (5.01)
		Chipanzee	1 (0.14)	1 (0.26)	0 (0.00)
		Human	21 (3.08)	5 (1.31)	16 (5.35)
	Order Lagomorpha	Rabbit	3 (0.44)	3 (0.78)	0 (0.00)
	Order Artiodactyla	Ox/cow	35 (5.14)	11 (2.88)	24 (8.02)
		Pig	5 (0.73)	1 (0.26)	4 (1.33)
		Hedgehog	1 (0.14)	0 (0.00)	1 (0.33)
		Giraffe	3 (0.44)	3 (0.78)	0 (0.00)
		Sheep	2 (0.29)	1 (0.26)	1 (0.33)
Total	15	40	681 (99.92)	382 (56.09)	299 (43.90)
Invertebrates	Invertebrata	Invertebrates	46 (36.22)	26 (40.00)	20 (32.25)
	Phylum Porifera	Porifers	1 (0.78)	0 (0.00)	1 (1.61)
		Marine sponges	2 (1.57)	2 (3.07)	0 (0.00)
	Phylum Cnidaria	Jellyfish	1 (0.78)	0 (0.00)	1 (1.61)
		Cnidarian	2 (1.57)	0 (0.00)	2 (3.22)
	Phylum Platyhelminthes	Flatworms	1 (0.78)	0 (0.00)	1 (1.61)
	Class Trematoda	Planarians	1 (0.78)	1 (1.53)	0 (0.00)
	*Class Cestoda*	Tapeworm	1 (0.78)	1 (1.53)	0 (0.00)
	Phylum Nematoda	Nematelmints	1 (0.78)	0 (0.00)	1 (1.61)
	Phylum Annelida	Earthworm	4 (3.14)	1 (1.53)	3 (4.83)
	Phylum Mollusca	Clam	2 (1.57)	0 (0.00)	2 (3.22)
	Class Cephalopoda	Cephalopod	1 (0.78)	0 (0.00)	1 (1.61)
		Octopus	1 (0.78)	0 (0.00)	1 (1.61)
	Class Gastropoda	Garden snail	6 (4.72)	1 (1.53)	5 (8.06)
		Snail	7 (5.51)	0 (0.00)	7 (11.29)
	Phylum Arthropoda	Arthropods	1 (0.78)	0 (0.00)	1 (1.61)
	Class Arachnida	Arachnids	1 (0.78)	1 (1.53)	0 (0.00)
		Spider	1 (0.78)	1 (1.53)	0 (0.00)
		Scorpion	6 (4.72)	4 (6.15)	2 (3.22)
	Class Chilopoda	Centipede	1 (0.78)	0 (0.00)	1 (1.61)
	Class Insecta	Insects	15 (11.81)	7 (10.76)	8 (12.90)
		Cockroach	1 (0.78)	1 (1.53)	0 (0.00)
		Mosquito	20 (15.74)	19 (29.23)	1 (1.61)
		Butterfly	1 (0.78)	0 (0.00)	1 (1.61)
		Caterpillar	2 (1.57)	0 (0.00)	2 (3.22)
		Cricket	1 (0.78)	0 (0.00)	1 (1.61)
Total	15	26	127 (99.84)	65 (51.18)	62 (48.81)
Other designations					
Vertebrates			123 (79.87)	72 (78.26)	51 (82.25)
Carnivores			6 (3.89)	4 (4.34)	2 (3.22)
Oviparous			2 (1.29)	2 (2.17)	0 (0.00)
Herbivores			4 (2.59)	0 (0.00)	4 (6.45)
Rational animals			1 (0.64)	0 (0.00)	1 (1.61)
Domestic animals			1 (0.64)	0 (0.00)	1 (1.61)
Harmful animals			1 (0.64)	1 (1.08)	0 (0.00)
Marine animals			7 (4.54)	6 (6.52)	1 (1.61)
Aquatic animals			3 (1.94)	2 (2.17)	1 (1.61)
Terrestrial animals			4 (2.59)	4 (4.34)	0 (0.00)
Wild animals			1 (0.64)	1 (1.08)	0 (0.00)
Germs			1 (0.64)	0 (0.00)	1 (1.61)
Total 13			154 (99.91)	92 (60.00)	62 (40.00)
Other groups					
Dengue virus			1 (6.25)	1 (25.00)	0 (0.00)
Bacteria			11 (68.75)	1 (25.00)	10 (83.33)
Protozoa			1 (6.25)	1 (25.00)	0 (0.00)
Fungi			2 (12.50)	0 (0.00)	2 (16.66)
Mosses			1 (6.25)	1 (25.00)	0 (0.00)
Total 5			16 (100.00)	4 (25.00)	12 (75.00)

^a^We consider the designation by local nomenclature

**Table 2:** Mean percentage (%) of animals cited by students as the most studied in science/biology classes, for Fundamental II and Ensino Médio

Grade	Fish		Amphibians		Reptiles		Birds		Mammals	
Ensino Fundamental	Rural	Urban	Rural	Urban	Rural	Urban	Rural	Urban	Rural	Urban
6	0.07	0.08	0.01	0.04	0.12	0.12	0.10	0.10	0.48	0.68
7	0.16	0.12	0.00	0.05	0.06	0.07	0.09	0.14	0.48	0.71
8	0.09	0.09	0.14	0.08	0.20	0.27	0.15	0.10	0.80	0.70
9	0.09	0.00	0.04	0.21	0.16	0.19	0.06	0.16	0.66	0.7
General mean	0.10	0.12	0.04	0.09	0.13	0.17	0.10	0.12	0.59	0.69
Ensino Médio										
1	0.08	0.19	0.16	0.07	0.30	0.23	0.14	0.19	0.74	0.95
2	0.07	0.13	0.12	0.11	0.22	0.26	0.17	0.09	0.79	0.63
3	0.16	0.19	0.29	0.09	0.27	0.14	0.13	0.07	0.79	0.57
General mean	0.09	0.16	0.17	0.09	0.27	0.22	0.15	0.14	0.77	0.79

Among the highest citation frequencies for all the groups of animals cited by the students were citations for the generic names “vertebrates,” “invertebrates,” “mammals,” “birds,” “reptiles,” “amphibians,” and “fish.” The last name accounted for 90% of the citations for that group, with the remaining 10% being citations for three specific fish (see Table 1).

Among the highest citation frequencies for mammals were those targeted at carnivores, such as “lion,” “jaguar,” “tiger,” and “bear,” for urban students, and the domestic “dog” and “cat,” for rural students. Other mammalian orders stood out for the frequencies of animals cited, especially “elephant” (Order Proboscidea), “whale” (Order Cetacea), “bat” (Order Chiroptera), and “rat” (Order Rodentia) for urban students, and “ox/cow” (Order Artiodactyla), “horse” (Order Perissodactyla), and “monkey” and “human” (Order Primates), for rural students (Table 1).

Among the birds cited, only “hawk” (Order Falconiformes) stood out from the others for its high frequency. Among reptiles, “snake” (Order Squamata) stood out for accounting for 60% of the citations for the group. Among amphibians, the citation frequency for “toad” (Order Anura) was around 55% of all citations for the group for both levels of schooling and for rural/urban groups.

Among invertebrates, the citation frequencies for “insects” and “mosquito” (Class Insecta) stood out. Among “other groups” of living organisms cited, the citation frequency for “bacteria” by rural students stood out, accounting for 83.33% of the citations in this category (Table 1).

In general, about 60% of the students stated that vertebrates of the region were addressed in their science/biology classes they experienced in their schooling processes (Table 3). Among these animals, the greatest richness and diversity was for mammals followed by birds for both urban (*n* = 38 mammals, *H*′ = 2.615; *n* = 20 birds, *H*′ = 2.457) and rural (*n* = 32 mammals, *H*′ = 2.821; *n* = 21 birds; *H*′ = 2.685) students. High diversity was also observed for invertebrates, despite the low richness of citations of “species” compared to the other analyzed groups, for both urban (*n* = 12 invertebrates, *H*′ = 2.441) and rural (*n* = 16 invertebrates, *H*′ = 2.560) students (Table 4). The average number of citations for all groups was always higher for Ensino Médio (see Table 5).

**Table 3:** Approaches to vertebrates in the region under study in science/biology classes, as confirmed by survey participants

Variables of responses by students	General	Urban	Rural
Yes	523 (60.18)	280 (58.94)	243 (61.67)
No	346 (39.81)	195 (41.05)	151 (38.32)
Total	869 (99.99)	475 (99.99)	394 (99.99)

**Table 4:** Frequencies of vertebrates (%) indicated by students as region native to the region, studied in Science/Biology classes

Group	Taxon	Animal^a^	General	Urban	Rural
Animal	Kingdom Animalia	Animals	1 (11.11)	1 (25.00)	0 (00.00)
Caatinga Animals		Caatinga Animals	1 (11.11)	1 (25.00)	0 (00.00)
Domestic animals		Domestic animals	1 (11.11)	1 (25.00)	0 (00.00)
Vertebrates	Subphylum Vertebrata	Vertebrates	6 (66.66)	1 (25.00)	5 (100.00)
Total	2	4	9 (99.99)	4 (44.44)	5 (55.55)
Pisces	Superclass Pisces	Fish	20 (74.07)	13 (81.25)	7 (63.63)
	Class Selachimorpha	Shark	3 (11.11)	3 (18.75)	0 (00.00)
	Order Perciformes	Tilapia	2 (7.40)	0 (00.00)	2 (18.18)
	Order Osteoglossiformes	Wolf fish	1 (3.70)	0 (00.00)	1 (9.09)
	Order Characiformes	Piaba	1 (3.70)	0 (00.00)	1 (9.09)
Total	5	5	27 (99.98)	16 (59.25)	11 (40.74)
Amphibians	Class Amphibia	Amphibians	1 (2.77)	0 (0.00)	1 (5.00)
	Order Anura	Toad	23 (63.88)	10 (62.50)	13 (65.00)
		Gia	2 (5.55)	0 (0.00)	2 (10.00)
		Frog	7 (19.44)	4 (25.00)	3 (15.00)
		Tree-frog	3 (8.33)	2 (12.50)	1 (5.00)
Total	2	5	36 (99.97)	16 (44.44)	20 (55.55)
Reptiles	Class Reptilia	Reptiles	2 (1.39)	1 (1.63)	1 (1.21)
	Order Squamata	Lizard	9 (6.29)	3 (4.91)	6 (7.31)
		Gecko	17 (11.88)	8 (13.11)	9 (10.97)
		Small Gecko	3 (2.09)	0 (0.00)	3 (3.65)
		Iguana	1 (0.69)	1 (1.63)	0 (0.00)
		Tegu lizard	6 (4.19)	2 (3.27)	4 (4.87)
		Chameleon	11 (7.69)	1 (1.63)	10 (12.19)
		Snake	74 (51.74)	35 (57.37)	39 (47.56)
	Order Crocodylia	Alligator	10 (6.99)	6 (9.83)	4 ((4.87)
		Crocodile	2 (1.39)	1 (1.63)	1 (1.21)
	Order Saurischia	Dinosaur	1 (0.69)	1 (1.63)	0 (0.00)
	Order Chelonia	Tortoise	4 (2.79)	0 (0.00)	4 (4.87)
		Water Tortoise	1 (0.69)	1 (1.63)	0 (0.00)
		Turtle	2 (1.39)	1 (1.63)	1 (1.21)
Total	5	14	143 (99.90)	61 (42.65)	82 (57.34)
Birds	Class Aves	Birds	43 (21.60)	29 (28.71)	14 (14.28)
	Order Columbiformes	Ground-dove	1 (0.50)	0 (0.00)	1 (1.02)
		Little dove	18 (9.04)	5 (4.95)	13 (13.26)
		Picazuro pigeon	7 (3.51)	4 (3.96)	3 (3.06)
		Dove	1 (0.50)	1 (0.99)	0 (0.00)
	Order Falconiformes	Southern Caracara	8 (4.02)	2 (1.98)	6 (6.12)
		Hawk	13 (6.53)	4 (3.96)	9 (9.18)
	Order Pelecaniformes	Heron	6 (3.01)	2 (1.98)	4 (4.08)
	Order Apodiformes	Hummingbird	5 (2.51)	5 (4.95)	0 (0.00)
	Order Passeriformes	Red-cowled Cardinal	2 (1.00)	1 (0.99)	1 (1.02)
		Canarian	2 (1.00)	1 (0.99)	1 (1.02)
		Sparrow	1 (0.50)	0 (0.00)	1 (1.02)
	Ordem Galliformes	Domestic Hen	17 (8.54)	10 (9.90)	7 (7.14)
		Guinea hen	1 (0.50)	0 (0.00)	1 (1.02)
	Order Accipitriformes	Vulture	17 (8.54)	10 (9.90)	7 (7.14)
	Order Psittaciformes	Parakeet	10 (5.02)	8 (7.92)	2 (2.04)
		Parrot	1 (0.50)	1 (0.99)	0 (0.00)
		Macaw	3 (1.50)	3 (2.97)	0 (0.00)
	Order Ciconiiformes	Stork	3 (1.50)	1 (0.99)	2 (2.04)
	Order Struthioniformes	Greater rhea	11 (5.52)	0 (0.00)	11 (11.22)
		Ostrich	1 (0.50)	0 (0.00)	1 (1.02)
	Order Anseriformes	Duck	4 (2.01)	2 (1.98)	2 (2.04)
		Goose	1 (0.50)	1 (0.99)	0 (0.00)
	Order Strigiformes	Owl	15 (7.53)	10 (9.90)	5 (5.10)
	Order Cariamiformes	Red-legged Seriema	1 (0.50)	0 (0.00)	1 (1.02)
	Ordem Tinamiformes	Small Tinamou	7 (3.51)	1 (0.99)	6 (6.12)
Total	15	26	199 (99.89)	101 (50.75)	98 (49.24)
Mammals	Class Mammalia	Mammals	5 (0.69)	1 (0.23)	4 (1.33)
	Order Carnivora	Dog	135 (18,75)	94 (22.38)	41 (13.66)
		Cat	110 (15.27)	78 (18.57)	32 (10.66)
		Lion	41 (5.69)	35 (8.33)	6 (2.00)
		Wolf	3 (0.41)	1 (0.23)	2 (0.66)
		Bear	12 (1.66)	12 (2.85)	0 (0.00)
		Giant Panda	1 (0.13)	1 (0.23)	0 (0.00)
		Jaguar Onca	1 (0.13)	1 (0.23)	0 (0.00)
		Jaguar	22 (3.05)	15 (3.57)	7 (2.33)
		Wild cat	9 (1.25)	4 (0.95)	5 (1.66)
		Tiger	20 (2.77)	12 (2.85)	8 (2.66)
		Fox	25 (3.47)	7 (1.66)	18 (6.00)
		Short-eared Dog	1 (0.13)	0 (0.00)	1 (0.33)
	Order Proboscidea	Mammoth	1 (0.13)	0 (0.00)	1 (0.33)
		Elephant	1 (0.13)	1 (0.23)	0 (0.00)
	Order Artiodactyla	Ox/Cow	96 (13.33)	41 (9.76)	55 (18.33)
		Goat	12 (1.66)	4 (0.95)	8 (2.66)
		Sheep	11 (1.52)	2 (0.47)	9 (3.00)
		Ram	2 (0.27)	2 (0.47)	0 (0.00)
		Hipoppotamus	2 (0.27)	1 (0.23)	1 (0.33)
	Order Perissodactyla	Horse	75 (10.41)	44 (10.47)	31 (10.33)
		Donkey	12 (1.66)	4 (0.95)	8 (2.66)
		Pig	15 (2.08)	4 (0.95)	11 (3.66)
		Zebra	1 (0.13)	1 (0.23)	0 (0.00)
	Order Cetacea	Whale	2 (0.27)	2 (0.47)	0 (0.00)
		Dolphin	2 (0.27)	1 (0.23)	1 (0.33)
	Order Lagomorpha	Rabbit	10 (1.38)	8 (1.90)	2 (0.66)
	Order Cetartiodactyla	Deer	8 (1.11)	3 (0.71)	5 (1.66)
	Order Rodentia	Rat	14 (1.94)	11 (2.62)	3 (1.00)
	Order Pilosa	Anteater	5 (0.69)	3 (0.71)	2 (0.66)
	Order Cingulata	Armadillo	18 (2.50)	7 (1.66)	11 (3.66)
		Six-banded Armadillo	15 (2.08)	2 (0.47)	13 (4.33)
	Order Chiroptera	Bat	6 (0.83)	4 (0.95)	2 (0.66)
	Order Primates	Lion Tamarin	1 (0.13)	1 (0.23)	0 (0.00)
		Human	5 (0.69)	2 (0.47)	3 (1.00)
		Monkey	10 (1.38)	6 (1.42)	4 (1.33)
	Order Diprotodontia	Kangaroo	3 (0.41)	2 (0.47)	1 (0.33)
	Order Didelphimorphia	White-eared Opossun	4 (0.55)	1 (0.23)	3 (1.00)
		Brazilian common opossum	2 (0.27)	1 (0.23)	1 (0.33)
	Ordem Didelphidae	Opossum	2 (0.27)	1 (0.23)	1 (0.33)
Total	16	40	720 (99.76)	420 (58.33)	300 (41.66)
Invertebrates	Invertebrata	Invertebrates	1 (1.81)	0 (0.00)	1 (2.43)
	Phylum Porifera	Marine Sponge	1 (1.81)	1 (7.14)	0 (0.00)
	Phylum Annelida	Earthworm	7 (12.72)	1 (7.14)	6 (14.63)
	Phylum Mollusca	Slug	1 (1.81)	0 (0.00)	1 (2.43)
	Phylum Arthropoda				
	Class Arachnida	Spider	2 (3.63)	2 (14.28)	0 (0.00)
		Scorpion	1 (1.81)	1 (7.14)	0 (0.00)
	Class Insecta	Insects	2 (3.63)	1 (7.14)	1 (2.43)
		Cricket	3 (5.45)	0 (0.00)	3 (7.31)
		Grasshopper	1 (1.81)	0 (0.00)	1 (2.43)
		Gnat	2 (3.63)	0 (0.00)	2 (4.87)
		Wasp	2 (3.63)	0 (0.00)	2 (4.87)
		Fly	4 (7.27)	0 (0.00)	4 (9.75)
		Ants	5 (9.09)	2 (14.28)	3 (7.31)
		Beetles	5 (9.09)	1 (7.14)	4 (9.75)
		Cockroach	1 (1.81)	0 (0.00)	1 (2.43)
		Butterfly	2 (3.63)	1 (7.14)	1 (2.43)
		Caterpillar	6 (10.90)	1 (7.14)	5 (12.19)
		Mosquito	6 (10.90)	1 (7.14)	5 (12.19)
		Mantis	1 (1.81)	1 (7.14)	0 (0.00)
		Bee	1 (1.81)	0 (0.00)	1 (2.43)
	Phylum Echinodermata	Sea star	1 (1.81)	1 (7.14)	0 (0.00)
Total	7	21	55 (99.86)	14 (25.45)	41 (74.54)
Others	Human morphology	Human body	2 (66.66)	1 (100.00)	1 (50.00)
	Prokaryotes	Bacteria	1 (33.33)	0 (0.00)	1 (50.00)
Total	2	2	3 (99.99)	1 (33.33)	2 (66.66)

**Table 5:** Mean percentage (%) of animals cited as of the region of the study by students, by grade, in cycles of Fundamental II and Ensino Médio

Grade	Fish		Amphibians		Reptiles		Birds		Mammals	
Ensino Fundamental	Rural	Urban	Rural	Urban	Rural	Urban	Rural	Urban	Rural	Urban
6	0.01	0.00	0.02	0.14	0.10	0.14	0.08	0.17	0.40	0.97
7	0.03	0.05	0.00	0.17	0.18	0.17	0.03	0.24	0.80	1.01
8	0.05	0.02	0.00	0.08	0.18	0.08	0.31	0.18	0.46	0.60
9	0.00	0.00	0.09	0.02	0.17	0.02	0.33	0.15	0.90	0.5
General mean	0.02	0.02	0.03	0.10	0.16	0.10	0.18	0.18	0.64	0.78
Ensino Médio										
1	0.01	0.05	0.12	0.14	0.21	0.14	0.31	0.26	0.67	0.76
2	0.02	0.13	0.05	0.13	0.25	0.13	0.24	0.22	0.55	0.76
3	0.06	0.00	0.00	0.14	0.19	0.14	0.27	0.01	0.74	1.03
General mean	0.03	0.06	0.07	0.14	0.22	0.13	0.28	0.21	0.65	0.81

Among the local mammals cited as most studied by students in their schooling processes, the highest citation frequencies by urban students were the domestic carnivores “dog” and “cat,” followed by “lion,” “jaguar,” “tiger,” and “fox” while for rural students, it was the same except for “fox,” which was cited more frequently. Two other mammalian orders also stood out for their citation frequency: “ox/cow” (Order Artiodactyla), most frequently cited by rural students, and “horse” (Order Perissodactyla) by urban and rural students (see Table 4). For birds, the highest citation frequency was for the generic term “bird,” mainly by urban students; specific citations highlighted, mainly by rural students, were “dove” (Order Columbiformes) and “hawk” (Order Falconiformes), in addition to “chicken” (Order Galliformes), “vulture” (Order Accipitriformes), and “owl” (Order Strigiformes). Among the reptiles indicated as the most studied buy students, the highest citation frequency was for “snake,” followed by “gecko” and “chameleon” (all Order Squamata), with the latter being almost exclusively cited by rural students. For amphibians, the most cited were “toad” and “frog” (both Order Anura). For fish, citations were practically limited to the generic term “fish” (Table 4). Among the invertebrates cited as most studied by students, the highest frequency was for “earthworm” (Phylum Annelida) by rural students (Table 4).

When asked if the importance of wildlife conservation was discussed in the science/biology classes experienced, the answer was yes for 68.75% of Fundamental II and 66.25% of Ensino Médio urban students, and 58.90% of Fundamental II and 66.90% of Ensino Médio rural students (Table 6). Regarding conceptual understanding of “Conservation of Nature,” 40.48% of Fundamental II and 52.20% of Ensino Médio urban students chose the single conceptual alternative offered that was in line with Brazilian curricular guidelines, while 31.21% of Fundamental II and 50.00% of Ensino Médio rural students chose this option (Table 6).

**Table 6:** Approaches to wildlife conservation in science/biology classes*, and conceptual understand of “Conservation of Nature”**, by urban and rural students in cycles of Fundamental II and Ensino Médio

Contexts	Urban		Rural	
Variable 1*	Yes	No	Yes	No
Ensino Fundamental	253 (68.75)	115 (31.25)	185 (58.90)	129 (41.08)
Ensino Médio	106 (66.25)	54 (33.75)	99 (66.90)	49 (33.10)
Total	359	169	284	178
Variable 2**				
Ensino Fundamental	149 (40.48)	219 (59.51)	98 (31.21)	216 (68.79)
Ensino Médio	84 (52.50)	76 (47.50)	74 (50.00)	74 (50.00)
Total	233	295	172	290

## Discussion

The indication of mammals and birds as the most studied animals in the schooling processes experienced, as well as the most indicated as locally occurring, by the questioned students evidences a tendency of greater human interest in these animal groups, which reinforces the results of other studies [22–24]. This tendency may reflect the greater phylogenetic proximity of these animals with humans, or utilitarian, aesthetic, or conflictual factors, among others [8, 22, 25–30]. These factors, reinforced by the students’ greater interest in and curiosity about these animal groups, may influence their approach to formal education processes. In this sense, Lindemann-Matthies [26] noted that children, as well as adults, in all age groups at school are more interested in animals, especially large mammals.

For the other animal groups, the data revealed a tendency toward limited knowledge regarding diversity, with a predominance of citation frequencies being for generic terms such as “birds,” “reptiles,” “snakes,” “amphibians,” and “fish,” with no indication of the diversity that exists within each of these groups. This fact may reflect factors such as habitat and morphological aspects of these animals, but also, above all, little emphasis on the diversity of these animal groups in the education processes. Limitations in knowledge regarding the diversity of some animal groups have a significant impact on human alienation from animal conservation [22]. Thus, knowledge is considered a priority condition for the development of positive behaviors and attitudes [22], without which conservation efforts are not viable [30]. In the case of amphibians, for example, Tarrant et al. [30] highlighted that research with students in South Africa revealed that conceptual limitation with these animals is frequent, even among educators.

Mammals and birds were indicated as the most addressed local animals in the educational processes experienced. Among mammals, however, domestic animals (“dog” and “can”) were the most cited, followed by exotic animals (“lion,” “jaguar,” and “tiger”) and only one locally occurring animal (“fox”), which reflects a limited understanding of the local fauna. In addition to these, citations were also frequent for “ox/cow” (Order Artiodactyla) and “horse” (Order Perissodactyla). Among the birds mentioned, in addition to the domestic “chicken” (Order Galliformes), there was a predominance of birds that are the target of hunting or are involved in conflicts with local people, especially in the rural context. These results allow us to reiterate conclusions observed in previous studies, that in addition to the influence of phylogenetic proximity, human interest in animals rests on aesthetic and utilitarian factors, among others [8, 22, 25–30], reverberating, therefore, in the curricular approach practiced. In addition, our results, in part, are supported by curricular parameters for Brazilian basic education (Ensino Básico), which emphasize the relationship between the thematic blocks “Environment” and “Technological Resources,” the use of living beings as natural resources, examples of hunting and animal breeding, and problematic predatory initiatives, with a view to the development of conservationists [1, 2].

For the other animal groups indicated as the most studied by the research participants, there were limitations in their recognition of the diversity of animals cited, and thus of their ecological importance. This constraint limits the development of critical awareness in light of the importance of biodiversity conservation since, as pointed out by Orientações Curriculares Brasileiras (Brazilian Curriculum Guidelines) for basic education, knowledge of the diversity of life, as well as of aspects related to its conservation, must appear as fundamental aspects of education processes, from the perspective of developing critical awareness about relationships/interactions between humans and other life forms [1–5]. Among the objectives of biodiversity education, we highlight the relevance of broader diversification of known organisms, for which school education has an indispensable function [7].

In all of the situations analyzed, even when the approach was directed to vertebrates in the region under study, invertebrates were cited as animals studied in science/biology classes, despite the low richness of the citations when compared to other cited animal groups. The highest frequencies were for “insects,” “mosquitos” (Class Insecta), and “earthworm” (Phylum Annelida), reflecting, perhaps, greater contact between students and these animals, especially in the rural context. We also highlight, among other cited groups, a high citation frequency for “bacteria,” mostly by rural students. In all these cases, one may conclude, among other factors, that there is inattention and/or poor clarity about the distinction between “vertebrates,” “invertebrates,” and “unicellular beings.” Because it is an elementary aspect, and of utmost importance for subsequent learning with a view to the development of critical awareness in reading and understanding about life forms, it is worth reflecting on the role of schooling, especially biology education, in developing adequate recognition of animal categories. Converging with these considerations, the Orientações Curriculares Brasileiras for basic education emphasize the importance of knowing, from the initial grades of schooling, how to classify animals into groups, such as vertebrates and invertebrates, among others, with an emphasis on their ecological and evolutionary implications [1, 2]. Páramo and Galvis [6] emphasized that in some cases, children do not recognize animal categorizations. Reinforcing investments in early childhood education, therefore, are critical for appreciating wildlife in subsequent stages of schooling [22, 26, 31].

In practically all situations analyzed, the average number of citations of animals was higher at the end of Ensino Médio, which reflects, in our view, the cumulative effect of the curriculum experienced, contributing to the expansion of the repertoire of animals known by students. That is, according to the curricular guidelines for basic education in Brazil, the content about animals is expanded from one schooling cycle to another, aiming at the development of valorization of biodiversity and the preservation of environments. In other words, biological contents are repeatedly addressed in depth as the cycles of schooling advance [1, 2, 5], based on a cognitive-based curricular logic [32–36].

An approach emphasizing the importance of animal conservation was recognized in the educational processes experienced by the majority (almost 70%) of the students, which is an important finding. However, conceptual understanding of “Conservation of Nature” ranged from 31.21% for rural students in Ensino Fundamental II to 52.20% for urban students in Ensino Médio. This finding reflects, in a way, disagreement with Brazilian curriculum guidelines on the subject, which proposes that this topic should be approached, implicitly or explicitly, throughout all curricular practice in basic education [1–5], in addition to the more specific approach of it as a transverse (cross-cutting) theme “Environment,” in which the conceptual notion “Conservation of Nature” is explained as the use of natural resources in a “rational” way, seeking a good yield without exhausting capacity for “renewal” or its ability to be “self-sustaining”; and still adds, based on Brazilian law, “implies management, use with care and maintenance” [10, 11]. Such conceptual understanding has convergences with theoretical orientations of ecology [12–16]. Therefore, our results suggest the need for more theoretical investments in this conceptual understanding in formal educational processes, especially in the rural context where the aforementioned understanding is more critical. In short, efficiency in conceptual understanding of biodiversity and its conservation presupposes a rethinking of the curriculum from a more contextual, systemic, and interdisciplinary perspective [6, 9, 18].

## Final considerations

The indication of mammals and birds as the most studied animals in the educational processes experienced by students in science/biology classes, with emphasis on large, showy carnivores with utilitarian value, among other aspects, does not differ from the results of other approaches of research involving interactions between humans and other animals. That is to say, what occurs in school, in curricular terms, with regard to animals reflects what happens in the context of people’s lives.

In contrast, for other animal groups, there was a tendency for limited recognition of the diversity of species the students understand. This leads us to the conclusion that, for these animal groups, educational processes need to go further in terms of exploration of, in an emphatic way, aspects related to the diversity of these animal groups, with a view to developing critical awareness regarding their conservation.

The citation frequencies for invertebrates, in addition to bacteria, mostly by rural students, when the focus of the approach was on vertebrates allows us to conclude that, if not inattention of the investigated students, there is little clarity in distinguishing among “vertebrates,” “invertebrates,” and “unicellular beings.” This reflects the educational approaches experienced by the students and may have repercussions on future learning and understanding of life forms.

Although almost 70% of students stated that their schooling processes addressed the conservation of wildlife, which is an important result. However, nearly 70% of rural students in ciclo Fundamental II and 59% of the other urban and rural students did not express clarity in their conceptual understanding of “Conservation of Nature,” despite the emphasis given to this theme in the curricular guidelines for basic education in Brazil. Thus, we suggest that more theoretical investments be directed toward this conceptual dimension in educational process, especially for rural students, who express less clarity about this conceptual aspect of animal conservation.

## Data Availability

All data generated or analyzed during this research are included in this published article.

## Footnotes

1 We considered the notion of diversity as a measure of the variability of individuals in a given context, such as the vertebrate groups in the present study, considering the relative frequency for each cited animal. The more equitable the frequencies of citation of the animals in the group, the greater the diversity. We refer to the notion of “local diversity” when referring to the number of species in a restricted area of homogeneous habitat (RICKLEFS 2003).
